# Immunomodulatory effects of ovalbumin hydrolysates in a mouse model of food allergy

**DOI:** 10.1186/2045-7022-5-S3-P118

**Published:** 2015-03-30

**Authors:** Daniel Lozano-Ojalvo, Ivan López-Expósito, Elena Molina, Rosina López-Fandiño

**Affiliations:** 1 Institute of Food Science Research (CIAL, CSIC-UAM), Madrid, Spain

## Background

Peptides released from enzymatic hydrolysis of certain proteins could exert immumodulatory effects on disorders such as food allergy. Peptide-based vaccines, corresponding to T cell epitopes of particular allergens, may retain immunogenicity, while being of insufficient length to cross-link allergen specific IgE on the surface of effector cells to elicit an allergic response. Ovalbumin (OVA) hydrolysates are a rich source of biologically bioactive peptides, some of which may present stimulatory activities on immune functions. On the other hand, cell cultures derived from mouse models of food allergy have proved very helpful in the evaluation of the allergenic and immunomodulatory abilities of hydrolysates and pure peptides. The aim of this study was to evaluate OVA hydrolysates as allergenic immune modulators by using splenocyte cultures of OVA-sensitized mice. The final goal is the development of new forms of immunotherapy, safer and more effective than those using whole allergens.

## Methods

OVA was hydrolyzed with commercial enzymes (pepsin, Alcalase and Neutrase) and their products size-fractionated (3 kDa and 10 kDa). The hydrolysates and their fractions were characterized by SDS-PAGE, RP-HPLC and HPLC-MS/MS, and their IgE-binding properties were evaluated by inhibition ELISA using sera of egg-allergic patients. Balb/c mice were sensitized to OVA by the oral administration of the protein with cholera toxin. At the end point, mice splenocytes were isolated and cultured in presence of the hydrolysates and their fractions alone, or together with the sensitizing protein. The immunomodulatory effect of the hydrolysates was based on the cytokine profile, as determined by ELISA.

## Results

In splenocyte cultures incubated in presence of the hydrolysates and their fractions, a marker down-regulation of Th2-biased cytokines (IL-4, IL-5 and IL-13) was observed. When samples were stimulated with OVA, the 10 kDa-fractions showed a more pronounced reduction of Th2 cytokines, as compared with the parent hydrolysates. The levels of Th1-biased cytokines (IFN-γ and TNF-α) increased in the presence of the hydrolysates with Alcalase and Neutrase. The hydrolysates produced with Neutrase significantly increased the levels of regulatory IL-10, even in the presence of the allergen.

## Conclusion

Hydrolysates of OVA with Neutrase exhibited an effective immunomodulatory effect in the cytokine profile of OVA-sensitized mice splenocytes. This might represent a novel immunotherapeutical approach in the treatment of egg allergy.

